# Goat *PDGFRB*: unique mRNA expression profile in gonad and significant association between genetic variation and litter size

**DOI:** 10.1098/rsos.180805

**Published:** 2019-01-30

**Authors:** Wenjing Yang, Hailong Yan, Ke Wang, Yang Cui, Tong Zhou, Han Xu, Haijing Zhu, Jinwang Liu, Xianyong Lan, Lei Qu, Chuanying Pan, Enping Zhang

**Affiliations:** 1 College of Animal Science and Technology, Northwest A&F University, Yangling, Shaanxi 712100, People's Republic of China; 2 Shaanxi Provincial Engineering and Technology Research Center of Cashmere Goats, Yulin University, Yulin 719000, People's Republic of China; 3 Life Science Research Center, Yulin University, Yulin 719000, People's Republic of China

**Keywords:** cashmere goat, *PDGFRB* gene, expression profile, insertion/deletion (indel), litter size

## Abstract

*β-Type platelet-derived growth factor receptor* (*PDGFRB*) is a typical tyrosine kinase, as a candidate gene associated with reproduction. Its main roles include regulation of gonocytes (migration and proliferation) and of the cell cycle. The objectives of this study were to identify mRNA expression of the goat *PDGFRB* gene, as well as insertion/deletion (indel) variants and their association with litter size in 1122 healthy Shaanbei white cashmere goats. The results revealed that *PDGFRB* was widely expressed in all tested tissues, and the expression levels in testes at different developmental stages indicated a potential association with the mitosis-to-meiosis transition. Furthermore, the expression of *PDGFRB* was relatively higher in the ovary tissue of mothers of two lambs compared with mothers of single lamb. These results implied that *PDGFRB* was related to goat fertility. Meanwhile, two intronic indels, 5 bp (*n* = 501) and 10 bp (*n* = 1122), were identified. Statistical analysis revealed that only the 10 bp indel was associated with first-born litter size (*n* = 1122, *p* = 6.030 × 10^−5^), and that individuals of the genotype insertion/deletion had larger litter sizes than those of genotype insertion/insertion. Overall, these results indicated that the 10 bp indel of *PDGFRB* could be used in marker-assisted selection during goat genetic breeding.

## 1. Introduction

Since the beginning of the twenty-first century, the goat industry in China has developed rapidly and its share in the national economy has also increased year by year. During this time, litter size has been a low heritability trait that has received widespread attention. Currently, when compared with traditional breeding methods, marker-assisted selection (MAS) based on genetic variation has proved to be more efficient for improving economically relevant traits of low heritability [1,2]. However, crucial genetic variants in the genome that lead to excellent phenotypic traits need to be validated.

At present, numerous potential genetic variations that were associated with phenotypic traits have been revealed by using genome-wide sequencing and genome-wide association study (GWAS) [3–5]. Although the analysis of large amounts of data has produced multiple potential gene variants, only a few relevant experimental need to be verified [4–6]. In order to select the dominant gene mutations that affected phenotypic traits, a method of combining GWAS and MAS has been established [6,7]. In 2017, using GWAS, Liu *et al*. [8] and Qin *et al*. [9] found that a single-nucleotide polymorphism (SNP) of the *β-type platelet-derived growth factor receptor* (*PDGFRB*) gene was significantly associated with semen production traits in Chinese Holstein bulls, which has since been validated by a larger study. These results implied the feasibility of screening crucial genetic variations by combining different methods.

The *PDGFRB* gene encodes a receptor tyrosine kinase (PDGFRB), a transmembrane protein belonging to class III receptor tyrosine kinases (RTKs) [10,11]. *PDGFRB* plays a dominant role in the proliferation and migration of gonocytes. Furthermore, the inhibition of mice PDGFRB tyrosine kinase activity leads to a decrease in testicular size, delayed spermatogenesis and a drastic reduction in epididymal sperm count [12]. Alternatively, the combination of PDGFRB and its ligand is important for the activation of primordial follicles as they transition to the primary stage, and mutations in PDGFRB in mice are lethal prior to follicle development in the ovary [13–15]. Moreover, PDGFRB is not only involved in the synthesis of steroid hormones of mice, but also controlled the development of steroidogenic cells [16]. Alterations in the steroid hormone levels were associated with many types of infertility in both males and females, such as hypogonadism and polycystic ovary syndrome (PCOS) [16]. Meanwhile, as a growth factor, PDGFRB regulates the cell cycle and thus affects cell proliferation [17]. All in all, based on the above studies, these results strongly demonstrated that the *PDGFRB* gene is associated with mammalian reproduction.

So far, there have been no relevant reports regarding the function of the PDGFRB gene in Shaanbei white cashmere goats (SBWC) reproduction. Therefore, in this study, expression profiles of the *PGDFRB* gene in SBWC were initially assessed in different tissues (heart, liver, spleen, lung, kidney, testis, brain, skin and muscle), and at different developmental stages in the testis and the ovary of ewes of different litter size. Meanwhile, two intronic insertion/deletion (indel) variants of the intron of *PDGFRB* gene were identified, namely the 5 bp indel and the 10 bp indel. Importantly, sequencing found a novel 36 bp indel downstream of the 5 bp indel locus, which was separated by a 49 bp sequence. The relationship between these loci and litter size was evaluated in large groups of SBWC. These results not only extend the knowledge of goat *PDGFRB* genetic variation, but also provide the basis for MAS of goat molecular breeding.

## 2. Material and methods

### 2.1. Animal and sample collection

For RNA experiments, we harvested nine tissues (heart, liver, spleen, lung, kidney, testis, brain, skin and muscle) from three-week-old ram (*n* = 3 per group). Moreover, a total of 16 testis tissues at 0, 3 days, one, two, three, four, six and eight weeks, and ovaries from seven ewes that had different litter size were also sampled. All tissues were stored at −80°C until used for analysis. For DNA experimentation, 1122 healthy female SBWC were randomly sampled and their first-born litter size was recorded. The feeding conditions were similar for all goats.

### 2.2. Total RNA isolation and cDNA synthesis

According to the manufacturer's instructions, total RNA was extracted from collected tissue samples using Trizol Reagent (Takara, Dalian, China). The purity and concentration of RNA were checked with a Nanodrop 2000 Spectrophotometer and 1% agarose gel electrophoresis. Samples could be used for reverse transcription when the OD_260_ nm/OD_280_ nm ratio of RNA was in the range of 1.8–2.0. Subsequently, a Prime Script™ RT Reagent kit (Takara) and universal primers were used in reverse transcription, and negative controls were prepared under the same conditions. The concentration of PrimeScript Reverse Transcriptase was 200 U µl^−1^, and the reaction volume and amount of RNA were 20 µl and 1 µg, respectively. The resultant cDNA was preserved at −20°C.

### 2.3. *PDGFRB* mRNA expression profiles analysis

According to the information on goat *PDGFRB* mRNA (Ref-seq accession XM_018050163.1 and XM_018050162.1), quantitative real-time PCR (qRT-PCR) primers were designed in Primer-BLAST of the NCBI (table 1). However, the primers needed to cover different exons to ensure cDNA amplification. The expression profiles of *PDGFRB* were analysed in a StepOnePlus^™^ Real-Time PCR System (Applied Biosystems, MA, USA) using cDNA from the three-week-old tissues. In addition, the expression profiles of testis tissue at different times (0, 3 days, one, two, three, four, six and eight weeks) and ovarian tissue of ewes of different litter size (MSL, mothers of single lamb; MTL, mothers of two lambs) were also examined.

**Table 1.** The qRT-PCR and amplification PCR primer sequences of the goat *PDGFRB* gene. Note: TD, touch-down PCR.

primer name	primer sequences (5′-3′)	sizes (bp)	detection methods
*PDGFRB*-F	GAGTCGGTGGACTATGTGCC	189	qRT-PCR
*PDGFRB*-R	CTGGTAGCTGAAGCCCACAA		
*Stra8*-F	TCTCACACTCCTCCGTCACT	192	qRT-PCR
*Stra8*-R	ATGCCTGCAAGAGGATGGTC		
*GAPDH*-F	AAAGTGGACATCGTCGCCAT	116	internal control
*GAPDH*-R	CCGTTCTCTGCCTTGACTGT		
P1(*PDGFRB*-1F)	CCAGCTCAGGATGGGTCT	296	TD
P1(*PDGFRB*-1R)	CCAGCATGGGCACATAGTC		
P2(*PDGFRB*-2F)	ACCTGAATCTGTCTGGTGTGT	222	TD
P2(*PDGFRB*-2R)	CAGAAAGGGAAAGGGACATGC		
P3(*PDGFRB*-3F)	GCTGGGTGAGGGCTACAAAA	120	TD
P3(*PDGFRB*-3R)	AAACACCAGTGCGTCACAGT		

The 13 µl qRT-PCR reaction contained 5 µl cDNA (1/100 dilution), 0.5 µl of each primer, 6.75 µl 2 × SYBR^®^ Premix Ex Taq™ II and 0.25 µl ROX-dye (Takara). The *GAPDH* (*glyceraldehyde-3-phosphate dehydrogenase*) was used as an endogenous control for the normalization of expression levels of *PDGFRB* gene mRNA (table 1), and three replicates were performed. The amplification cycle was as follows: initial denaturation and activation of the polymerase for 5 min at 95°C for one cycle, followed by 40 cycles at 94°C for 30 s, 60°C for 30 s and then 72°C for 30 s. The final extension was for 10 min at 72°C. And, the 2^−ΔΔC_t_^ method was used to transform C_t_ values into normalized relative expression levels of mRNA.

### 2.4. Genomic DNA isolation and DNA pool construction

Blood samples were obtained from ear tissue of all 1122 SBWC. Genomic DNA was isolated as described by Lan *et al*. [18] and stored at −20°C. Genomic DNA concentration was quantified by Nanodrop 2000, and the working solution of each DNA sample was brought to 10 ng µl^−1^. Fifty DNA samples were randomly selected to construct genomic DNA pools for exploring the genetic variation of the *PDGFRB* gene in SBWC [19].

### 2.5. Indel identification and sequencing

Based on the goat (*Capra hircus*) gene sequence (GenBank accession NC_030814.1) and the NCBI SNP-database (https://www.ncbi.nlm.nih.gov/snp), three pairs of primers (table 1, P1–P3) were designed to amplify genomic DNA pools to explored genetic variation in the goat *PDGFRB* gene. PCR reactions were performed with touch-down (TD) PCR in a 13 µl volume, which contained 6.5 µl 2× mix, 0.5 µl of each primer, 0.5 µl 10 ng µl^−1^ genomic DNA and 5 µl ddH_2_O. The TD PCR reaction procedure was as follows: initial denaturation for 5 min at 95°C; followed by 18 cycles of denaturation for 30 s at 94°C; annealing for 30 s at 68°C (with a decrease of 1°C per cycle); extension for 30 s at 72°C; another 23 cycles of 30 s at 94°C, 30 s at 50°C and 2 min at 72°C; and a final extension for 10 min at 72°C, with subsequent cooling to 4°C [20]. The PCR products were detected by means of 3.5% agarose gel electrophoresis that stained with ethidium bromide and SensiCapture 12.3 (Peiqing JS-2012 auto-focus Gel Image Analyzing System, Shanghai, China). The specificity of PCR products was confirmed by sequencing when each pair of primers produced a single objective band.

### 2.6. Statistical analysis

Genotypic frequencies, allelic frequencies and linkage disequilibrium (LD) were calculated using the SHEsis program (http://analysis.bio-x.cn) [21]. The *χ*^2^-test was used to evaluate the Hardy–Weinberg equilibrium (HWE) [22]. Polymorphism information content (PIC) was calculated using online software (http://www.msrcall.com/Gdicall.aspx) [20]. Distribution differences for genotypic and allelic frequencies were analysed using the *χ*^2^-test or Fisher exact tests (when the minimum theoretical frequency was less than 5) in SPSS (version 19.0).

Association between the indel loci and first-born litter size was analysed with a general linear model: *Y_ijklm_* = *μ* + *S_i_* + HYS*_j_* + *P_k_* + *G*_l_+*e_ijklm_*, where *Y_ijklm_* was the phenotypic value of litter size, *μ* was the overall population mean, *S_i_* was the effect of lambing year, HYS*_j_* was the mean of the population, *P_k_* was the fixed effect of the parity, *G_l_* was the fixed effect of the genotype and *e_ijklm_* was the random error [23,24]. The lambing year and parity were not considered in the model, because the data of litter size used in this study were first-born litter size. Further analysis was performed with SPSS 19.0 software using Student's *t*-test (*t*-test), the data were rejected when *n* < 5. And, *p* < 0.05 was considered statistically significant.

## 3. Results

### 3.1. Tissue expression profiles of the goat *PDGFRB* gene

**Figure 1.**
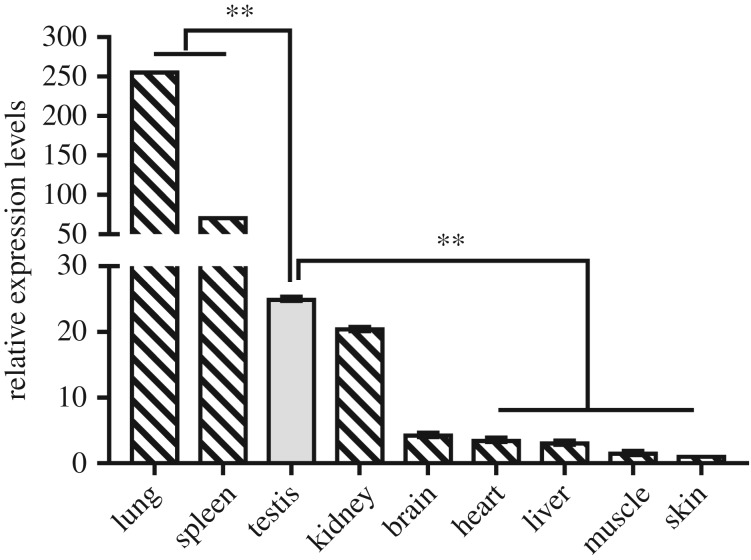
SBWC PDGFRB mRNA expression patterns detected by qRT-PCR. Different tissues of three-week-old SBWC. Data represent means ± s.e. (*n* = three samples of each tissues). *p < 0.05; **p < 0.01.

Expression profiles of the *PGDFRB* gene from different tissues at three weeks old were investigated. The results revealed that the *PDGFRB* gene was expressed in all tested tissues (heart, liver, spleen, lung, kidney, muscle, brain, skin and testis). Expression level was extremely high in the lung, which was followed by the spleen, and then the testis. Interestingly, the expression levels were significantly different between the testis and skin, muscle, liver, heart, spleen and lung (*p* < 0.01), but not for the brain or kidney (*p* > 0.05) (figure 1).

### 3.2. Goat *PDGFRB* gene expression profiles of gonads

**Figure 2.**
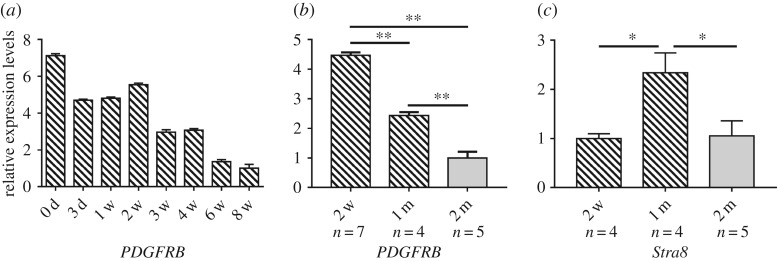
SBWC *PDGFRB* and *Stra8* mRNA expression patterns detected by qRT-PCR. (*a*) The mRNA expression profiles in goat testis at different developmental stages. (*b*) Expression of *PDGFRB* mRNA at mitosis (two weeks and one month, *n* = 7 and *n* = 4, respectively) and meiosis (two months, *n* = 5) in testis tissue. (*c*) Expression of *Stra8* mRNA at mitosis (two weeks and one month, *n* = 4 and *n* = 4, respectively) and meiosis (two months, *n* = 5) in testis. Data represent means ± s.e. *p < 0.05; **p < 0.01.

We examined the expression of the goat *PDGFRB* gene in testis collected at different developmental stages and the ovaries of ewes of different litter size. Results showed that the expression of *PDGFRB* was highest at 0 day, and the overall expression revealed a downward trend with the extension of time. Moreover, the mRNA expression of *PDGFRB* at six and eight weeks old was lower than that at other stages (figure 2*a*).

Studies have shown that in the Liaoning cashmere goat (the male parent of SBWC) enter the mitotic stage of spermatogenesis after birth, and the primary spermatocytes that initiate meiosis first occur in one month or so [25]. So, we compared the expression levels of *PDGFRB* gene in the testis during the mitotic periods of zero to two weeks and one month (*n* = 7 and *n* = 4, respectively) and the meiotic period of one to two months (*n* = 5) [26,27]. Compared to the meiotic stage, the expression of *PDGFRB* was significantly downregulated as mitosis shifted to meiosis (*p* < 0.01) (figure 2*b*). The expression level of *Stra8* (*stimulated by retinoic acid 8*), a marker gene for mammalian germ cell transition from mitosis to meiosis [28], was significantly upregulated during anaphase of mitosis (one month) (*p* < 0.05) (figure 2*c*). In short, these results indicated that the *PDGFRB* gene plays an important role in male reproduction.

**Figure 3.**
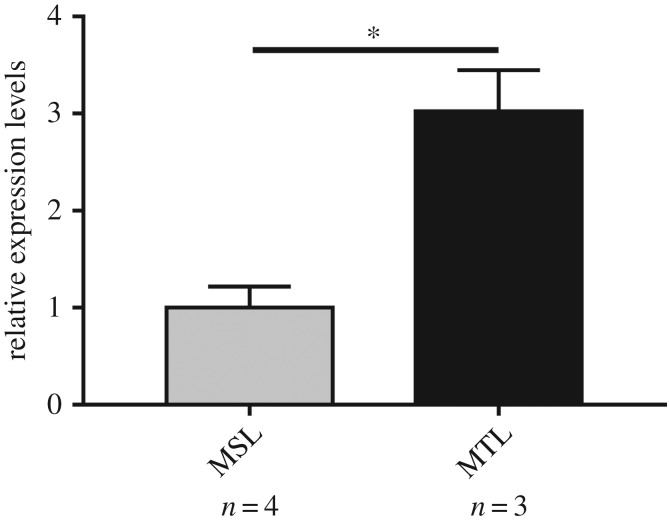
The mRNA expression profile of *PDGFRB* in SBWC ovary. Data represent means ± s.e. *p < 0.05; **p < 0.01.

The expression of *PDGFRB* gene in goat ovaries of ewes of different litter size showed that expression of the *PDGFRB* gene in MTL was significantly higher than that in MSL (*p* < 0.05) (figure 3). This proved that the goat *PDGFRB* gene was also essential for female reproduction.

### 3.3. Identification indel of the *PDGFRB* gene

**Figure 4.**
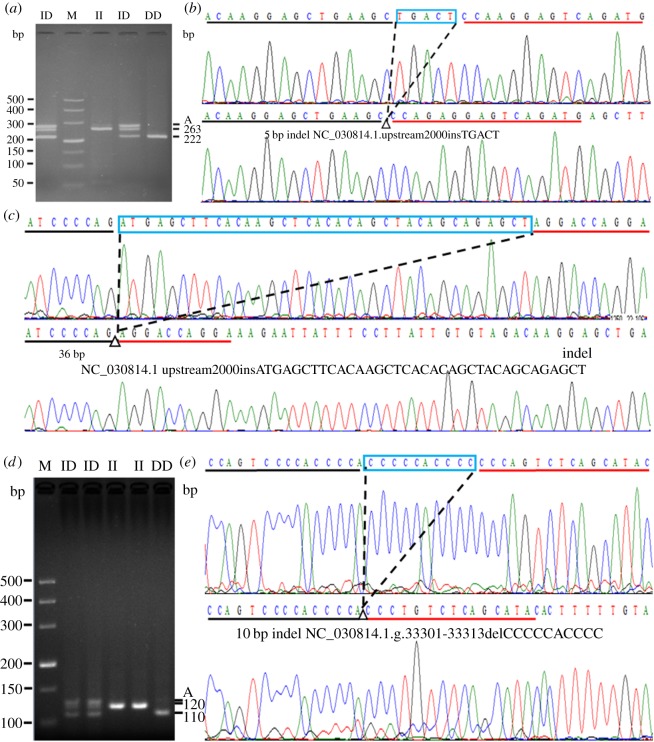
The electrophoresis diagrams and sequence diagrams of goat *PDGFRB* gene indel loci. (*a*,*b*) The 5 bp indel locus, (*a*,*c*) the 36 bp indel locus and (*d*,*e*) 10 bp indel locus. The letter A indicated a heteroduplex and the triangle symbol (Δ) indicated the location of the insertion/deletion (indel). The underlined nucleotides at the beginning of the sequences represent similarities between the ‘ins’ variant and ‘del’ variant.

In order to explore potential DNA markers for improving goat fertility, we next carried out an identification of indel variants. In this study, there were two pairs of primers that had genetic variation (table 1, P2–P3). Primer P3 amplified a fragment with an expected size, named 10 bp indel (NC_030814.1. g.33301-33313delCCCCCACCCC) (figure 4*d*,*e*). Reciprocally, the DNA product amplified by primer P2 did not match the fragment with an expected size (227/222 bp), and the individuals of 14 insertion/insertion (II) genotypes and 31 DD genotypes were randomly selected and sequenced. The results showed a novel 36 bp indel (NC_030814.1.upstream.2000insATGAGCTTCACAAGCTCACACAGCTACAGCAGAGCT) downstream of the expected 5 bp indel (NC_030814.1.upstream.2000insTGACT), which was separated by a 49 bp sequence (figure 4*a*–*c*). Both the 5 and 36 bp indels were inserted or absent at the same time from the sequencing diagram. These results suggested that the two indel loci may be linked.

### 3.4. Linkage disequilibrium analysis of the *PDGFRB* gene

**Figure 5.**
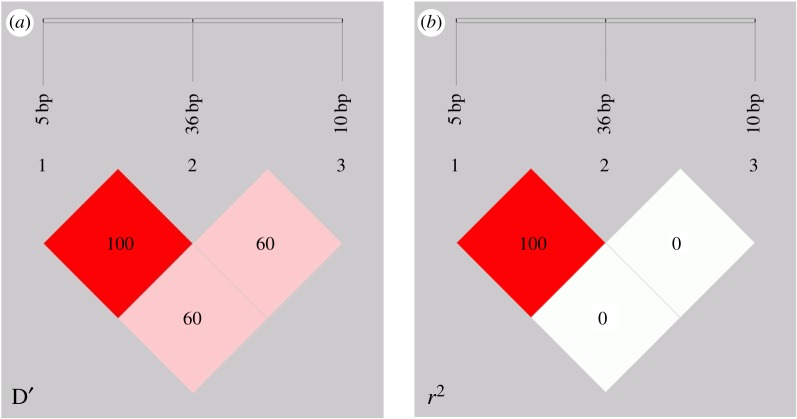
LD plot of the *PDGFRB* gene three indel loci in goat, 5 bp indel, 36 bp indel and 10 bp indel. (*a*) *D*′ value, (*b*) *r^2^* value.

LD of the 5, 36 and 10 bp indel in SBWC was analysed. As shown in figure 5 and table 2, for the 5 and 36 bp indel, the values of *D′* and *r*^2^ were 1.00 and 1.00, respectively (table 2 and figure 5). The *r*^2^ value was used as a pairwise measure of LD, which implied that the 5 bp indel and 36 bp indel were completely linked. In relative terms, there was no LD between these two indel and the 10 bp indel (*r*^2^ = 0.007) (table 2 and figure 5).

**Table 2.** *D′* and *r*^2^ values of pairwise LD of the *PDGFRB* gene in SBWC.

	*D'*		*r^2^*	
loci (bp)	36	10	36	10
5	1.00	0.606	1.000	0.007
36		0.606		0.007

### 3.5. Genetic parameter analysis of the indel variants

Next, the genotypes and allele frequencies, as well as the polymorphisms associated with the indel locus in the *PDGFRB* gene, were calculated. The distribution of genotypes was determined in SBWC by HWE analysis. For the 5 and 36 bp indel, the frequency of allele ‘D’ was highest (0.795), and the frequency of genotypes II, insertion/deletion (ID) and DD were 0.058, 0.293 and 0.649, respectively, in 501 individuals (table 3). At the 10 bp indel, only 3 ‘DD’ genotypes were detected in 1122 individuals, and the frequency of the allele ‘I’ was greatest (0.908) (table 3).

**Table 3.** Genotypes, alleles, He, Ne, PIC and HWE analysis the indel within the *PDGFRB* gene in SBWC. Note: P2, 5 bp and 36 bp indel; P3, 10 bp indel; SBWC, Shaanbei white cashmere goat; HWE, Hardy–Weinberg equilibrium; Ho, homozygosity; He, heterozygosity; Ne, effective allele numbers; PIC, polymorphism information content.

					population parameters			
loci	sizes (*N*)	genotype frequencies	allele frequencies	HWE *p*-values	Ho	He	Ne	PIC
P2	501	II (29, 0.058)	I (0.205)	*p* > 0.05	0.674	0.326	1.484	0.273
		ID (147, 0.293)	D (0.795)					
		DD (325, 0.649)						
P3	1122	II (919, 0.819)	I (0.908)	*p* > 0.05	0.833	0.167	1.201	0.153
		ID (200, 0.178)	D (0.092)					
		DD (3, 0.003)						

The *χ*^2^-test also showed that the genotype frequencies of these loci (5, 36 and 10 bp) in the SBWC population were in accordance with the HWE (*p* > 0.05) (table 3). Additionally, gene homozygosity (Ho), heterozygosity (He), effective allele numbers (Ne) and PIC of these indel loci of *PDGFRB* gene were calculated. The values of PIC at these loci were 0.273 and 0.153, respectively, indicating that they were polymorphic in SBWC (table 3). Among them, the 10 bp indel was close to low genetic diversity (PIC = 0.153) (table 3).

### 3.6. Analysis of association with first-born litter size in SBWC

In the SBWC population, we analysed the relationship between the indel loci of *PDGFRB* gene and reproduction (first-born litter size) in ewes. There was no significant correlation between the 5 and 36 bp indel and first-born litter size among 0–501 samples that were selected randomly from the whole population (*χ*^2^-test, *p* > 0.05) (table 4). Interestingly, the 10 bp indel was consistently associated with the first-born litter size in 1122 individuals (Fisher's exact test, *p* < 0.05), except for 100 individuals at first (table 5). Moreover, genotype distributions were significantly different between the different lambing groups of goats (*p* < 0.01) (table 6). Association analysis also revealed that individuals of genotype ID outperformed genotype II individuals (*p* < 0.01) (table 7).

**Table 4.** Genotype distribution between mothers of litter size of 5 and 36 bp indel in SBWC. Note: *χ*^2^ test.

	mothers of single lamb			mothers of multi-lamb (≥2)			
numbers	II	ID	DD	II	ID	DD	*p*-values
100	3	17	30	4	12	34	0.534
200	7	29	62	7	27	68	0.874
300	13	37	98	13	49	90	0.375
400	15	52	135	14	68	116	0.168
501	15	70	167	14	77	158	0.741

**Table 5.** Genotype distribution between mothers of litter size of 10 bp indel in SBWC. Note: Fisher exact test. Italics indicate *p* < 0.05.

	mothers of single lamb			mothers of multi-lamb (≥2)			
numbers	II	ID	DD	II	ID	DD	*p*-values
100	43	7	0	35	15	0	0.09
200	93	7	0	78	22	0	*0.004*
300	139	13	0	119	29	0	*0.007*
400	184	18	0	165	33	0	*0.024*
500	231	21	0	212	36	0	*0.035*
600	271	31	0	231	6 7	0	*3.580 × 10^−5^*
800	352	51	1	313	81	1	*0.004*
1000	445	57	1	380	115	2	*7.699 × 10^−7^*
1122	532	84	1	387	116	2	*6.030 × 10^−5^*

**Table 6.** Genotype distribution between mothers of single lamb, two lambs and three lambs of 10 bp indel in SBWC. Note: Fisher exact test. Italics indicate *p* < 0.05.

		genotypes			genotype frequencies			
types	sample sizes	II	ID	DD	II	ID	DD	Fisher value *p-*value
mothers of single lamb	617	532	84	1	0.862	0.136	0.002	*Fisher = 19.423* *p = 4.447 × 10^−4^*
mothers of two lambs	492	376	114	2	0.764	0.232	0.004	
mothers of three lambs	13	11	2	0	0.846	0.154	0

**Table 7.** Relationship between the 10 bp indel of *PDGFRB* gene and litter size of first born in SBWC (LSM^a^ ± s.e.) (*p* < 0.05/*p* < 0.01). Note: *t*-test, genotype DD (*n* = 3) less than 5, we rejected these data. Cells with different letters (a, b/A, B) differed significantly (*p* < 0.05/*p* < 0.01). Italics indicate *p* < 0.05.

	observed genotypes (LSM^a^ ± s.e.)		
Traits	II (922)	ID (204)	*p*-values
litter size	1.43^B^ ± 0.02	1.59^A^ ± 0.04	*1.103 × 10^−4^*

## 4. Discussion

To date, numerous studies have shown that *PDGFRB* not only participates in testicular development and affects the differentiation of gonocytes, but is also involved in the development of ovarian follicles [12–14]. These observations suggested that the *PDGFRB* gene could affect fertility. Previous studies have not reported on the expression profile of *PDGFRB* gene or the association between the *PDGFRB* indel locus and first-born litter size in SBWC.

Initially, the expression profile of the *PDGFRB* gene of SBWC was determined in this study, and the results showed it to be widely expressed in various organs (figure 1). It is important to note, however, that *PDGFRB* is reported to be associated with gonocytes proliferation and migration [12]. The expression of *PDGFRB* in testis tissues at different developmental stages was also detected, and we found this decreased progressively with development (figure 2*a*). The expression level of *PDGFRB* was highest at 0 days, and it was lowest at six and eight weeks (figure 2*a*). The spermatogonia mature from gonocytes that migrate to the basal membrane, a process which establishes and maintains spermatogenesis in the mature murine testis [12]. Furthermore, the number of PDGFRB-positive cells located in the centre of the seminiferous tubules decreases progressively after birth, they are found on the basal membrane at postnatal 5 (P5), and PDGFRB activity was drastically reduced after P5 [29]. At about a month of age, most of goat gonocytes have differentiated into spermatogonia and are attached to the basal membrane [30]. In Liaoning cashmere goats, spermatogonia gradually proliferate through mitosis since birth, and meiosis is initiated at one month [25]. We, therefore, speculated similar characteristics for this period in SBWC, and combined individuals of 0, 3 days, one, two, three and four weeks as the mitotic period (two weeks and one month), similarly the data of six and eight weeks were considered as the meiotic period (two months). Our results illustrated that the expression level of *PDGFRB* in SBWC during meiosis was significantly lower than that in the mitotic period (*p* < 0.01) (figure 2*b*). Moreover, we also tested the expression level of *Stra8* (*stimulated by retinoic acid 8*) at the same time points as *PDGFRB*. Retinoic acid (RA) regulates mouse spermatogonia cell differentiation as well as meiotic initiation through the target gene *Stra8*, and *Stra8* was also a marker gene for the germ cell transition from mitosis to meiosis in other mammals, such as rats, humans and goats, etc. [28,31,32]. As shown in figure 2*c*, *Stra8* was significantly upregulated at anaphase of mitosis (one month, *p* < 0.05) (figure 2*c*). Overall, these results revealed that the *PDGFRB* gene was essential for the production of spermatogonia and was associated with goat male reproduction.

Previous studies have also found that *PDGFRB* had a crucial role in female reproduction. Schmahl *et al*. [16] showed that mouse *PDGFRB* was involved in controlling the synthesis of steroid hormones in the ovary. Moreover, Brito *et al*. [15] identified the expression of *PDGFRB* in goat ovarian follicles, which is associated with follicular development. From our current study, figure 3 shows that the expression of the *PDGFRB* gene in MTL was significantly higher than that in MSL (*p* < 0.05). These findings highlight the role of *PDGFRB* in female reproduction. This was important because, with the development of society and the improvement of breeding techniques, female livestock commonly outnumber males. The purpose of our study was to screen genetic variation, which could help to implement MAS in goat breeding. The indel variants could be used as a type of genetic variation with important functions, compared with the SNPs, which had the advantages of convenient detection and remarkable effects [20]. Therefore, we studied and analysed the indel variants of *PDGFRB* in 1122 SBWC.

In this study, we found that there were three insertion/deletions (indels) in the upstream 2000 bp and the 22nd intron regions of the *PDGFRB* gene in goats, which were the 5 bp indel, 36 bp indel and 10 bp indel, respectively. These indel loci were consistent with the prediction by NCBI, expected for the 36 bp indel. Not only that, but the 36 bp indel was located downstream of the 5 bp indel, and they may have complete LD (table 2 and figure 4). Both indel loci had three genotypes (II, ID and DD), and were consistent with HWE (*p* > 0.05) (table 3).

Subsequently, the relationship between indel loci and first-born litter size was analysed. In order to make the result more reliable, we adopted a new strategy. In the whole population, 100 individuals were randomly taken as a subset, and different subsets were randomly selected and accumulated for analysis. Interestingly, the 10 bp indel was always associated with first-born litter size in 1122 individuals (*p* < 0.01), except for 100 individuals (table 5). Meanwhile, Fisher's exact test showed a significant correlation of genotype distribution and litter size within the different lambing groups of goats (*p* = 4.447 × 10^−4^) (table 6). Importantly, there were only three individuals with genotype DD (*n* < 5), so we rejected these data and compared the relationship between individuals with genotype II and ID by *t*-test. There was a significant difference in first-born litter size between genotype II and ID (*p* < 0.01), with the mutant (ID) having larger litters than the wild-type (II) (table 7). These results implied that ‘D’ was a dominant allele in the SBWC population, and that this 10 bp indel had great potential for application in breeding. Unfortunately, the 5 bp indel and 36 bp indel show no association between 0 and 501 individuals and first-born litter size (*p* > 0.05) (table 4).

**Figure 6.**
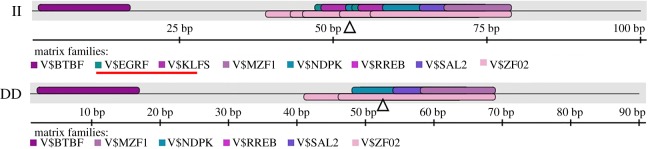
The prediction of transcription factor binding sites in intron of *PDGFRB*. The triangle symbol (Δ) indicated location of the indel. The red line indicated different transcription factor.

Although the 10 bp indel locus was located in intron 22, several studies have found that the intronic variations of the gene could affect transcription through transcription factors [26,27,33]. Van Laere *et al*. [34] found that the variation in intron 3 of *IGF2* impacted the expression in postnatal muscle, which controlled phenotypic variation, such as muscle growth and fat deposition. Therefore, in order to investigate why the 10 bp indel affected first-born litter size, we predicted the transcription factor binding sites in the regions where indel occurred using the online software Genomatix MatInspector (http://www.genomatix.de/) [35]. The results revealed that there were different transcription factors between genotypes II and DD as follows, namely *KLFs* (*Kruppel-like factors*) and *EGRF* (*epidermal growth factor receptor*) (figure 6). This discovery provided a possibility that two factors influence goat litter size. Among them, *KLF* was involved in cellular proliferation, survival and differentiation. Several *KLFs* participate in oestradiol signalling in the normal reproductive system, thereby regulating reproductive development and function [36]. Meanwhile, *EGRF* also regulates cell cycle progression, thereby affecting cell proliferation [37]. This suggests that *KLFs* and *EGRF* may be associated with reproduction and affected litter size.

Alternatively, some intronic variations may have LD to mutations with known phenotypes [38]. Such as, Gansmo *et al*. [39] found that the 40 bp indel in the mouse MDM2 (mouse double minute 2 homologue) gene promoter P1 was completely LD with an SNP, and this indel locus had a positive correlation with a reduced risk of endometrial cancer. Following these findings, the further study was needed regarding the way in which the 10 bp intronic of the goat *PDGFRB* gene affects the phenotype (first-born litter size).

## 5. Conclusion

To summarize, *PDGFRB* gene mRNA was expressed in all tested tissues (heart, liver, spleen, lung, kidney, testis, brain, skin and muscle) and the expression levels in the testis decreased with developmental age. In addition, the expression of *PDGFRB* was relatively higher in the ovary of MTL compared with those of MSL. Two novel intronic indels, 5 bp and 10 bp, were identified and the latter was significantly associated with first-born litter size (*p* < 0.001). These results provide the basis for MAS of goat molecular breeding.

## Supplementary Material

Supplementary Material-Figure of qPCR

## Supplementary Material

Supplementary Material-Genotype data of the indel loci

## Data accessibility

The data supporting the present study have been uploaded as part of the manuscript and electronic supplementary material.
