# Supplementary material for: Goat PDGFRB: unique mRNA expression profile in gonad and significant association between genetic variation and litter size
Source: R Soc Open Sci. 2019 Jan 30;6(1):180805. doi: 10.1098/rsos.180805 (PMC6366220; doi:10.1098/rsos.180805)
Supplement: Supplementary Material-Figure of qPCR [file rsos180805supp1.docx]

Goat *PDGFRB*: unique mRNA expression profile in gonad and significant association between genetic variation and litter size

Wenjing Yang^*^, Hailong Yan^*^, Ke Wang, Yang Cui, Tong Zhou, Han Xu, Haijing Zhu, Jinwang Liu, Xianyong Lan, Lei Qu, Chuanying Pan^**^, Enping Zhang^**^

* These authors contributed equally to this work.

** Corresponding authors: Chuanying Pan and Enping Zhang.

qRT-PCR validation:

For SYBR Green I, Ct of the no-template control (NTC):

*PDGFRB*: Undetermined

*GAPDH*: 34.77280553

It hinted that the qPCR system (Applied Biosystems, Massachusetts, USA) has no problem, and could be used for follow-up experiments.

**
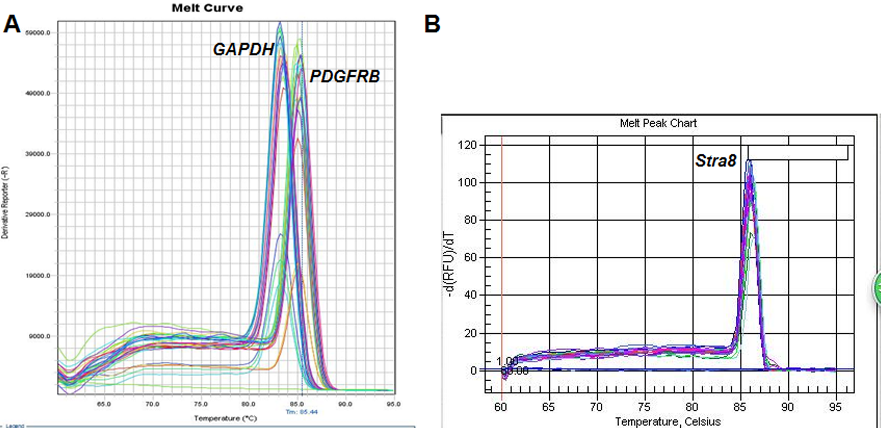
**

**Figure S1.** The melt curve. (A) *PDGFRB* and *GAPDH* genes. (B) *Stra8* gene.


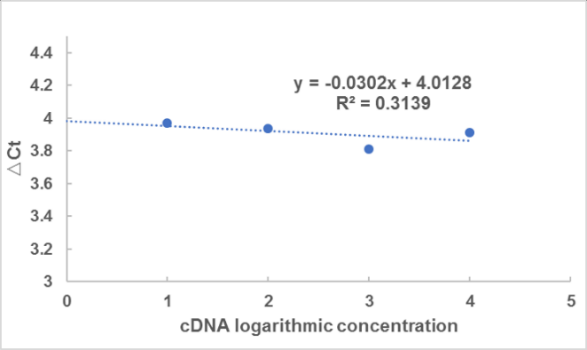


**Figure S2.** Validation of the 2^−ΔΔCt^ method. One cDNA sample was subjected to 10x gradient dilution as a template for amplification of *PDGFRB* and *GAPDH* genes, and they amplified by qRT-PCR. The curve of the relation between ΔCt and cDNA logarithmic concentration for *PDGFRB* and *GAPDH* genes. The absolute value of the line slope was 0.0302, which was close to 0, indicating that the amplification efficiency of the target gene and the internal control gene was the same, and the data could be analyzed by the qRT-PCR 2^-ΔΔCt^ method.
